# Left ventricular thrombus formation in myocardial infarction is associated with altered left ventricular blood flow energetics

**DOI:** 10.1093/ehjci/jey121

**Published:** 2018-08-22

**Authors:** Pankaj Garg, Rob J van der Geest, Peter P Swoboda, Saul Crandon, Graham J Fent, James R J Foley, Laura E Dobson, Tarique Al Musa, Sebastian Onciul, Sethumadhavan Vijayan, Pei G Chew, Louise A E Brown, Malenka Bissell, Mariëlla E C J Hassell, Robin Nijveldt, Mohammed S M Elbaz, Jos J M Westenberg, Erica Dall'Armellina, John P Greenwood, Sven Plein

**Affiliations:** 1 Division of Biomedical Imaging, LICAMM, University of Leeds, Leeds, UK; 2 The Department of Radiology, Leiden University Medical Center, Postalzone C2-S, RC Leiden, The Netherlands; 3 Radboudumc, Department of Cardiology, Geert Grooteplein Zuid 10, GA Nijmegen, The Netherlands

**Keywords:** thrombosis, myocardial infarction, magnetic resonance imaging, fluid dynamics, flow imaging

## Abstract

**Aims:**

The main aim of this study was to characterize changes in the left ventricular (LV) blood flow kinetic energy (KE) using four-dimensional (4D) flow cardiovascular magnetic resonance imaging (CMR) in patients with myocardial infarction (MI) with/without LV thrombus (LVT).

**Methods and results:**

This is a prospective cohort study of 108 subjects [controls = 40, MI patients without LVT (LVT− = 36), and MI patients with LVT (LVT+ = 32)]. All underwent CMR including whole-heart 4D flow. LV blood flow KE wall calculated using the formula: $KE=\frac{1}{2}\;\rho_{blood}\;.\;V_{voxel}\;.\;v^{2}$, where *ρ* = density, *V* = volume, *v* = velocity, and was indexed to LV end-diastolic volume. Patient with MI had significantly lower LV KE components than controls (*P* < 0.05). LVT+ and LVT− patients had comparable infarct size and apical regional wall motion score (*P* > 0.05). The relative drop in A-wave KE from mid-ventricle to apex and the proportion of in-plane KE were higher in patients with LVT+ compared with LVT− (87 ± 9% vs. 78 ± 14%, *P* = 0.02; 40 ± 5% vs. 36 ± 7%, *P* = 0.04, respectively). The time difference of peak E-wave KE demonstrated a significant rise between the two groups (LVT−: 38 ± 38 ms vs. LVT+: 62 ± 56 ms, *P* = 0.04). In logistic-regression, the relative drop in A-wave KE (beta = 11.5, *P* = 0.002) demonstrated the strongest association with LVT.

**Conclusion:**

Patients with MI have reduced global LV flow KE. Additionally, MI patients with LVT have significantly reduced and delayed wash-in of the LV. The relative drop of distal intra-ventricular A-wave KE, which represents the distal late-diastolic wash-in of the LV, is most strongly associated with the presence of LVT.

## Introduction

Left ventricular (LV) thrombus (LVT) remains a life-threatening complication of myocardial infarction (MI), being associated with a five-fold increased risk of systemic embolism.^1^ The risk for LVT is greater with anterior MI, low ejection fraction (EF), LV aneurysms, and apical akinesis or dyskinesis,^1^^,^^2^ but LVT formation can also be found in patients with smaller infarcts, inferior infarcts, and only mild to moderate LV systolic dysfunction.^3^

The development of LVT is a complex process involving substrates of the Virchow's triad: disturbance of flow (stasis or turbulence), hypercoagulability, and endothelial injury/dysfunction. Early echocardiographic studies have demonstrated that abnormal flow patterns are associated with LVT.^4–6^ However, comprehensive insight into flow changes in post-MI patients with LVT is lacking, partly because tests capable of examining the complex 3D intra-cavity flow have not been available in the past.

The development of four-dimensional (4D) flow cardiovascular magnetic resonance imaging (CMR) now allows mapping and quantification of intra-cavity LV flow kinetic energy (KE).^7–12^ LV blood flow KE appears to be reduced in patients with heart failure,^13^ and has the potential to provide new mechanistic insights into the pathophysiology of LVT formation in patients with ischaemic cardiomyopathy by detecting specific signatures of flow disturbance associated with LV flow stasis in LVT.

The aim of this study was to use 4D flow CMR to map LV flow KE and characterize flow changes in patients with MI associated ischaemic cardiomyopathy with and without LVT. We hypothesized that patients with LVT show a re-distribution of LV flow KE resulting in reduced wash-in and wash-out of the LV. Furthermore, we aimed to investigate if LV flow KE mapping parameters are better associated with presence of LVT than the traditional risk factors for the development of LVT.

## Methods

### Study population

**Figure 1:**
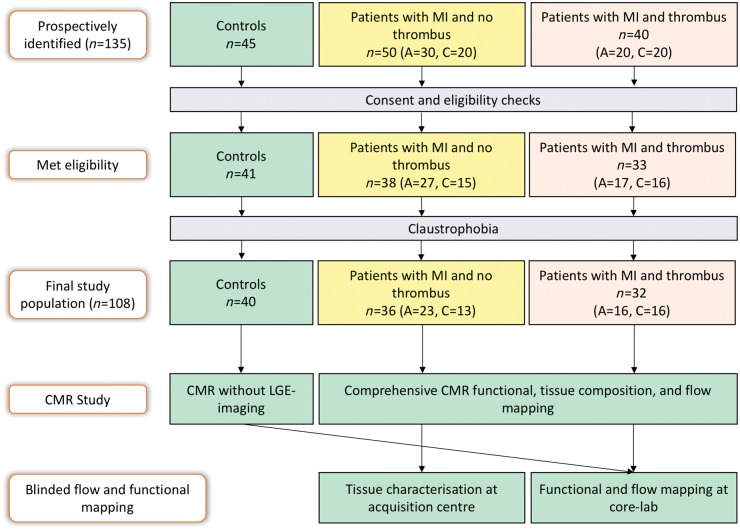
Study design. A, acute reperfused ST-elevation myocardial infarction; C, chronic myocardial infarction; CMR, cardiovascular magnetic resonance imaging; LGE, late gadolinium enhancement imaging; MI, myocardial infarction.

This was a prospective cohort study of patients with MI and matched controls (*Figure 1*). Controls were recruited from two centres (Leeds and Leiden). They had no history or symptoms of cardiovascular disease, were on no cardiovascular or other relevant medication and had no contraindications to CMR. MI patients were recruited in Leeds and included both acute ST-elevation MI (STEMI) and chronic MI patients (MI > 3 months). Because of the relatively low incidence of LVT, we identified patients with LVT from routine clinical echocardiography lab and clinical CMR lists between January 2015 and April 2017 and in parallel recruited age and gender matched patients with MI but without LVT. Patients identified for this study were offered research CMR bolt-on scans immediately after clinical CMR or full protocol CMR if identified from echocardiography lab. All patients presenting to our CMR lab gave prospective consent prior to their clinical scans for the bolt-on research protocol.

The inclusion criteria for acute STEMI patients were: first-time acute STEMI revascularized by primary percutaneous coronary intervention within 12 h of onset of chest pain. Acute STEMI patients were scheduled for CMR imaging within 72 h of indexed presentation. The inclusion criteria for chronic MI patients were: previous history of MI and presence of scar on late gadolinium enhancement (LGE) imaging. Exclusion criteria for all included the following: cardiomyopathy, atrial fibrillation, haemodynamic instability, and any contraindications to CMR.

### Ethical approval

The study protocol was approved by the National Research Ethics Service (12/YH/0169) in the United Kingdom and the institutional Medical Ethical Committee (P11.136) in Leiden, The Netherlands. The study complied with the Declaration of Helsinki and all patients gave written informed consent.

### CMR examination

All controls and patients underwent CMR imaging on identical 1.5 T systems at the two study sites (Ingenia, Philips, Best, The Netherlands) with 28-channel coils and digitization of the magnetic resonance signal in the receiver coil.

### CMR protocol and image acquisition

The CMR protocol included survey, cines, early gadolinium enhancement imaging, LGE imaging, and at the end 4D flow CMR.^14^

### 4D flow acquisition

For 4D flow, the field-of-view was planned in the trans-axial plane ensuring full cardiac coverage by adjusting the number of slices. A free-breathing, non-respiratory navigated, Echo-Planer Imaging (EPI)- accelerated 4D flow sequence was used.^14^ 4D flow data reconstruction and error corrections are detailed in the Supplementary data online, *Document S1*.

### Image analysis

All images were evaluated offline using in-house developed research software (MASS; Version 2017-EXP, Leiden University Medical Center, Leiden, The Netherlands). For functional and flow analysis, anonymised cine/4D flow CMR data were shared with the core lab at Leiden (*Figure 1*) and tissue characterisation was done at the main clinical acquisition site (Leeds) and remained blinded to core lab functional/flow analysis. Methods of analysis are descripted in the Supplementary data online, *Document S1*.

### KE mapping

**Figure 2:**
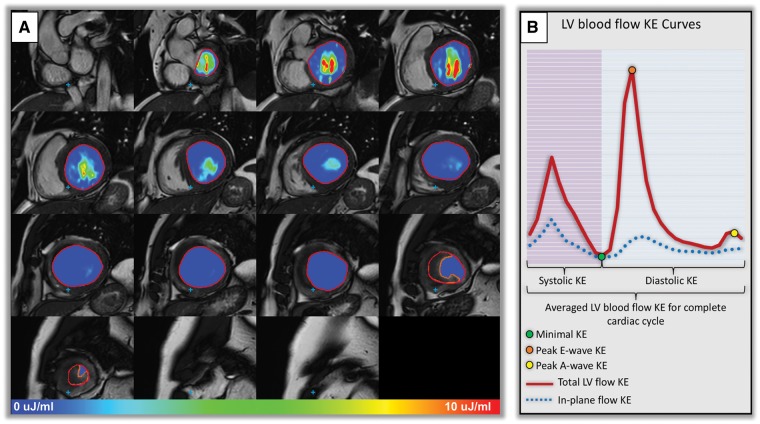
(*A*) Left ventricular short-axis endocardial segmentation in patient with LV thrombus. Intra-cavity thrombus was manually contoured (orange contour) to avoid under-estimation of LV KE parameters. Intra-cavity KE of blood is demonstrated at peak late ventricular filling (peak A-wave). (*B*) Illustration of KE curve demonstrating majority of the KE parameters studied.

LV was contoured manually in the images of the short-axis cine acquisition (*Figure 2A*). Correction for translational and rotational misalignment between the short-axis cine and the 4D flow CMR acquisition was performed using automated image registration as previously described.^15^

For calculation of LV blood flow KE parameters, the LV volumetric mesh was resliced into short-axis sections of 2 mm thickness and pixel spacing equal to the original reconstructed pixel size of the short-axis cine acquisition (1.0–1.2 mm). For each volumetric element (voxel) the KE was computed as $KE=\frac{1}{2}\;\rho_{blood}\;.\;V_{voxel}\;.\;v^{2}$, with $\rho_{blood}$ being the density of blood (1.06 g/cm^3^), $V_{voxel}$ the voxel volume, and v the velocity magnitude. For each phase, the total KE within the LV was obtained by summation of the KE of every voxel. In addition, the KE was computed for basal, mid and apical LV level by dividing the LV into equal thirds. Similarly, the in-plane component of KE (i.e. the short-axis plane) was computed, by taking for v the magnitude of the in-plane component of velocity. KE parameters were normalized to the LV end-diastolic volume and reported in μJ/mL (KEi_EDV_).

In patients with LVT, LVT was excluded from KE mapping to reduce under-estimation of LV flowing blood KE (*Figure 2A*). The global, wash-in, and wash-out components of the LV KE that were mapped are described in *Figure 2B*. During diastolic filling, the flow velocity and its associated KE of early (E-wave) and late (A-wave) filling decrease from base to apex. In this study, this drop has been measured as a relative drop in percentage from each level (base to mid-ventricle; mid-ventricle to apex) for both E-wave flow KE and A-wave flow KE. Higher relative-drop of LV in-flow KE signifies reduced wash-in of the LV.

### In-plane KE

The in-plane KE is the sum of all KE in the *x*-*y* direction, in the short-axis LV from base to apex. In this study, the in-plane KE is represented as a percentage of the total LV KE. This parameter was computed mainly to better understand the in-plane flow dynamics within the LV cavity.

### Time difference

We also computed the time difference (TD) to peak early mitral in-flow velocity (E-wave) from the base of the LV to mid-ventricle. This transit time or TD should be higher if the mitral valve propagation velocity (*V*p), as measured by M-mode echocardiography is lower. Hence, the transit time of the peak KE from base to mid-ventricle, described as the TD in this study, may represent a novel marker of delayed filling.

A detailed description of the CMR protocol, pulse sequences, and the intra-/inter-observer reproducibility test are given in the Supplementary data online, *Document S1*.

### Statistical analysis

Statistical analysis was performed using IBM SPSS^®^ Statistics 23.0. Quantitative parameters are presented as mean ± standard deviation or median and interquartile ranges, where appropriate. Demographic comparisons were performed with *post hoc* analysis of variance (ANOVA) with Bonferroni corrections. Imaging data was handled as non-parametric. Step-wise multivariate logistic regression was used for clinical, functional, and KE parameters with statistical significance from one-way analysis (*P* < 0.1). Diagnostic performance tests were done using the receiver-operator characteristic. To avoid collinearity issues within volumetric parameters, only LV EF was included in the multivariate analysis. A *P*-value <0.05 was considered statistically significant.

Sample size calculations are described in the Supplementary data online, *Document S1*.

## Results

### Demographic characteristics

We identified 135 subjects for this study, 23 did not meet the eligibility criteria and 4 were claustrophobic. Hence, 108 subjects completed the study (*Figure 1*). These included 40 controls (Leiden = 13, Leeds = 27), 36 LVT− patients and 32 LVT+ patients. From the 32 LVT+ patients, 5 LVT+ patients (16%) were identified at echocardiography lab and the rest were identified at the CMR lab. Patients on anti-coagulation had already been diagnosed with LVT by echocardiography.

All subjects had comparable heart rates (*P* > 0.05) (*Table 1*). Heart failure status was comparable between the two patient groups. Patients with LVT were more likely to have diabetes (*P* = 0.01) and to be on anti-coagulation than patients without LVT (*P* = 0.04).

**Table 1:** Study demographics (study population = 108)

	Younger controls (*n* = 24)	Age-matched controls (*n* = 16)	LVT− (*n* = 36)	LVT+ (*n* = 32)	*P*-value^a^	*P*-value^b^	*P*-value^c^
Baseline characteristics							
Age (years)	30 ± 10	57 ± 7	60 ± 9	61 ± 13	<0.01	0.7	1
Sex (female)	7	8	8	3	0.2	0.3	0.4
Body surface area (m²)	1.9 ± 0.2	1.8 ± 0.2	1.9 ± 0.2	2 ± 0.2	0.2	0.03	0.67
Smoker	0	0	23	13		<0.01	0.07
Hypertension	0	0	8	9		0.02	1
Hypercholesterolaemia	0	0	16	8		<0.01	0.14
Diabetes	0	0	2	8		0.48	0.01
Baseline clinical parameters							
Systolic BP (mmHg)			131 ± 34	137 ± 16			0.5
Heart rate (b.p.m.)	64 ± 7	65 ± 15	65.5 ± 9	66 ± 12	1	0.10	0.3
KC 1			33	25			0.12
KC 2			3	7			0.12
Medical therapy at the time of recruitment							
ACE-inhibitor			35	23			0.11
HMG-CoA reductase inhibitors			35	20			0.07
β-blockers			35	23			0.11
Aspirin			35	20			0.07
Anti-coagulation			1	4			0.04
Blood results							
Haemoglobin (g/dL)			144 ± 14	141 ± 12			0.99
eGFR			83.7 ± 9	77 ± 17			0.08
C-reactive protein (mg/L)			36 ± 38	24 ± 22			0.55
HBA1c (mmol/mol)			41.5 ± 11	47 ± 18			0.28

Data are presented as mean ± standard deviation or count (*n*).

BP, blood pressure; CAD, coronary artery disease; KC, heart failure Killip class.

a Younger controls vs. older age-matched to patient controls.

b Age-matched controls vs. LVT−.

c LVT− vs. LVT+.

### Baseline CMR

**Figure 3:**
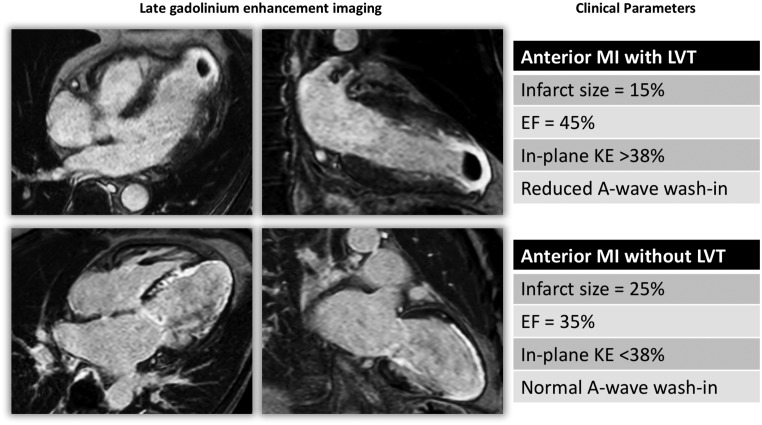
Late gadolinium enhancement imaging of two case examples from the study population with anterior MI. Infarct characteristics alone did not differentiate the two cases for LVT. Left ventricular KE flow analysis demonstrated rise in in-plane, rotational KE, and reduced A-wave wash-in of the LV. EF, ejection fraction; KE, kinetic energy; LVT, left ventricular thrombus.

Patients with LVT were more likely to have anterior MI than those without LVT (87% vs. 61%, *P* = 0.01). Infarct size was comparable between patients with/without LVT (*P* = 0.6) (*Figure 3* and *Table 2*). Also, for all four apical segments, scar transmurality was not different between patients with/without LVT (*P* > 0.05) (Supplementary data online, Table S4). Apical RWM-abnormality score showed a lower trend in LVT+ vs. LVT− but did not achieve statistical significance (*P* = 0.055). EF was significantly lower in patients with LVT and end-diastolic/end-systolic volumes and mass were significantly increased in patients with LVT compared with patients without LVT.

**Table 2:** Left ventricular baseline volumetric and haemodynamic study

	Younger controls (*n* = 24)	Age-matched controls (*n* = 16)	LVT− (*n* = 36)	LVT+ (*n* = 32)	*P*-value^a^	*P*-value^b^	*P*-value^c^
Volumetric assessment							
Anterior infarction (*n*)			22	28			0.01
Infarct size (% of LV)			21 ± 14	24 ± 12			0.6
Apical RWMA score			3 ± 1	4 ± 0.5			0.055
Stroke volume (mL)	105 ± 30	93 ± 28	77 ± 26	75 ± 31	<0.01	0.06	0.42
Ejection fraction (%)	62 ± 7	64 ± 5	44 ± 11	32 ± 17	0.13	<0.01	<0.01
LVEDVi (mL/m^2^)	91 ± 19	75 ± 21	91 ± 16	100 ± 43	<0.01	<0.01	0.01
LVESVi (mL/m^2^)	35 ± 11	27 ± 6	52 ± 15	67 ± 41	<0.01	<0.01	<0.01
LVMi (g/m^2^)	52 ± 15	49 ± 9	56 ± 13	68 ± 21	0.44	0.03	<0.01
Haemodynamic assessment							
E-wave velocity (cm/s)^d^	59 ± 44	48 ± 22	26 ± 9	27 ± 10	0.08	<0.01	0.66
A-wave velocity (cm/s)^d^	32 ± 19	34 ± 13	24 ± 8	24 ± 8	0.65	<0.01	0.21
*E*/*A* ratio	2 ± 0.6	1.3 ± 0.4	1.3 ± 0.7	1.2 ± 0.8	0.09	0.71	0.71
MDT (ms)	147 ± 35	140 ± 26	146 ± 30	137 ± 42	0.7	0.4	0.81
Mitral regurgitation (mL)	0 ± 0.01	0 ± 0.02	3 ± 4	4 ± 3	0.33	0.01	0.09

Data are presented as median ± interquartile range or count (*n*). LV measurements are indexed to body surface area.

LVEDVi, left ventricular end-diastolic volume (indexed); LVESVi, left ventricular end-systolic volume (indexed); LVMi, left ventricular mass (indexed); MDT, mitral deceleration time; MV, mitral valve; RWMA, regional-wall motion abnormality score (1 = normal, 2 = hypokinaesia, 3 = akinetic, 4 = diskinetic).

a Younger controls vs. older age-matched to patient controls.

b Age-matched controls vs. LVT−.

c LVT− vs. LVT+.

d These peak inflow velocities are average peak velocities for the full mitral annular flow.

### Haemodynamic analysis

Patients with infarct were more likely to have mitral regurgitation (MR) (*P* = 0.01). However, MR was similar in both patient groups (*P* = 0.09). No other mitral in-flow diastolic function parameter demonstrated any significant difference between the two MI groups.

### Thrombus characteristics

In the 36 patients recruited with LVT, 26 (72%) patients had mural thrombus, 6 (17%) had mobile thrombus and 10 (27%) had protruding thrombus. Average indexed volume of thrombus was 4.9 ± 10 mL/m^2^. Thrombus characteristics and its association to flow mapping are detailed in the Supplementary data online, Table S5.

### KE mapping

**Figure 4:**
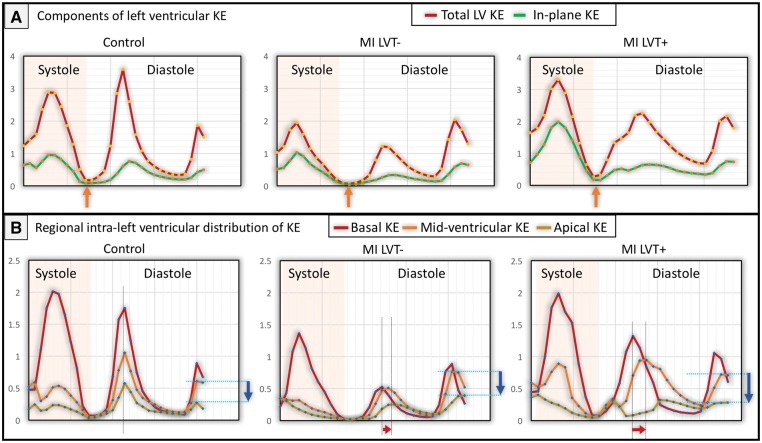
LV flow component KE curves in different study subjects. (*A*) KE curves for total and in-plane LV KE. The minimal KE is marked by orange arrows. (*B*) Regional KE curves for base, mid, and apex of the LV. Time differences to peak E-wave KE propagation also demonstrated progressive increase (gapes between dotted lines marked by red arrows). Blue arrows point to drop in A-wave KE from mid-ventricle to apex. KE, kinetic energy; LV, left ventricle; LVT−, patients without LV thrombus; LVT+, patients with LV thrombus; MI, myocardial infarction.

**Figure 5:**
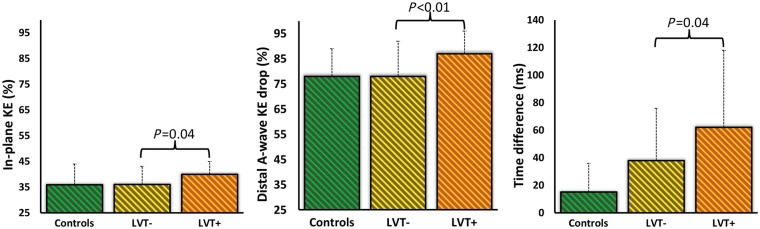
Bar graph of the main LV blood flow kinetic energy parameters which demonstrated significant changes in patients with/without thrombus (whiskers: interquartile range). KE, kinetic energy; LVT−, patients without LV thrombus; LVT+, patients with LV thrombus.

LV KEi_EDV_ averaged over the complete cardiac cycle was significantly lower in both patient groups vs. healthy controls (*P* < 0.05) (*Figure 4*). Similarly, average systolic KEi_EDV_ and peak E-wave KEi_EDV_ were significantly lower in patients than in age-matched controls (*Table 3*). Peak late filling (A-wave) KEi_EDV_ was not different between the three groups (*P* > 0.05). The proportion of in-plane KE of the LV was not different between controls and LVT− patients (*P* = 0.82). However, patients with LVT demonstrated a significantly higher proportion of in-plane KE vs. LVT− (40% vs. 36%, *P* = 0.02) (*Figure 5*).

**Table 3:** Detailed mapping of left ventricular kinetic energy

	Younger controls (*n* = 24)	Age-matched controls (*n* = 16)	LVT−	LVT+	*P*-value^a^	*P*-value^b^	*P*-value^c^
Global LV kinetic energy							
LV^d^	10 ± 4	8.3 ± 1.5	6.3 ± 2	5.6 ± 2	0.63	0.01	0.19
Minimal^d^	1 ± 0.4	0.9 ± 0.5	0.7 ± 0.56	0.8 ± 0.61	0.64	0.07	0.19
Systolic^d^	10 ± 3	9 ± 4	6.6 ± 2	6.7 ± 3	0.98	<0.01	0.65
Diastolic^d^	10 ± 4	8 ± 2	5.9 ± 3	5.4 ± 2	0.47	0.07	0.31
Peak E-wave^d^	25 ± 13	22 ± 12	12.4 ± 7	10.8 ± 8	0.01	<0.01	0.54
Peak A-wave^d^	9 ± 5	13 ± 10	11 ± 5	9.5 ± 5	0.01	0.15	0.08
In-plane KE (%)	33 ± 10	37 ± 6	36 ± 7	40 ± 5	0.27	0.82	0.02
Relative KE drop from base to apex (%)							
E-wave (B→M)	60 ± 18	52 ± 12	56 ± 16	65 ± 27	0.18	0.42	0.22
A-wave (B→M)	69 ± 12	68 ± 2	64 ± 17	60 ± 33	0.36	0.69	0.08
E-wave (M→A)	88 ± 8	89 ± 5	89 ± 9	89 ± 10	0.44	0.7	0.38
A-wave (M→A)	78 ± 10	78 ± 11	78 ± 14	87 ± 9	0.34	0.27	<0.01

Data are presented as median ± interquartile range or count (*n*). Unit of normalized kinetic energy: μJ/mL.

B→M, base to mid-ventricle; KE, kinetic energy; M→A, mid-ventricle to apex.

a Younger controls vs. older age-matched to patient controls.

b Age-matched controls vs. LVT−.

c LVT− vs. LVT+.

d KEi_EDV_ variables (μJ/mL).

The relative drop of proximal and distal, intra-ventricular E-wave KE, did not differ across the three groups (*P* > 0.05) (*Table 3*). Peak A-wave KE drop from mid-ventricle to apex was not different in controls and LVT− patients (*P* = 0.69). However, LVT+ patients demonstrated a significantly higher drop in A-wave KE from mid to apex when compared with LVT− patients (87% vs. 78%, *P* < 0.01) (*Figure 5*).

The TD of peak E-wave KE propagation from base to mid-ventricle demonstrated rise between all the four groups of subjects—younger controls = 14 ± 15 ms vs. age-matched older controls = 18 ± 28 ms (*P* = 0.52); age-matched older controls = 18 ± 28 ms vs. LVT− = 38 ± 38 ms (*P* = 0.07); LVT− = 38 ± 38 ms vs. LVT+ = 62 ± 56 ms (*P* = 0.04) with an overall ANOVA between groups of <0.001) (*Figure 5*).

### Logistic regression

**Figure 6:**
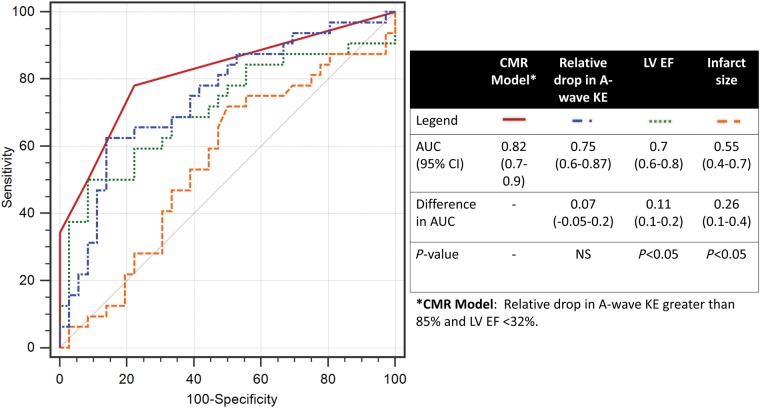
Receiver operating curve analysis for the presence of LVT.

In univariate analysis, the following parameters were associated with the presence of LVT: history of diabetes, anterior MI, EF, drop of A-wave KE from mid-ventricle to apex, proportion of LV in-plane KE, and TD of peak early-filling (E-wave) KE from base to mid-ventricle (*Table 4*). In multivariate analysis, only distal drop of A-wave KE (beta = 11.5, *P* = 0.002) and EF (beta = −0.08, *P* = 0.01) demonstrated independent association with LVT. A combined CMR model of EF and relative drop in A-wave KE demonstrated significantly larger area under the curve than LV EF [difference in AUC = 0.11, 95% confidence interval (CI) 0.1–0.23; *P* = 0.02] and infarct size (difference in AUC = 0.26, 95% CI 0.1–0.4; *P* = 0.02) (*Figure 6*).

**Table 4:** Logistic regression analysis of variables which influence presence of LVT

	Univariate			Multivariate		
	Beta	SD	*P*-value	Beta	SD	*P*-value
DM	−1.8	0.8	0.03			0.14
Anterior MI	−1.5	0.6	0.02			0.13
Ejection fraction	−0.81	0.03	<0.01	−**0.08**	**0.03**	**0.01**
Distal A-wave KE drop	8.4	2.9	<0.01	**11.5**	**3.7**	**0.002**
In-plane KE	0.13	0.06	0.02			0.19
Time difference	0.011	0.006	0.04			0.74

Bold results are highlight independent predictors in the regression model.

DM, diabetes mellitus; KE, kinetic energy; MI, myocardial infarction.

### Intra-/inter-observer reliability

Global LV KE parameters demonstrated very low bias (intra: average 2%; inter: average 4%) and good precision (intra: −16% to −20%; inter: −21% to −13%). Inter-rater reliability of main KE parameters thresholds were good (in-plane KE >37%; weighted-kappa = 1, distal A-wave KE drop >85%; weighted-kappa = 0.63, and TD from base to mid >31 ms; weighted-kappa = 0.67). Comprehensive results for individual parameters can be found in the Supplementary data online, *Document S1*.

## Discussion

This study provides mechanistic insights into intra-cavity LV flow disturbances in MI patients with and without LVT. Firstly, we demonstrate that global LV KEi_EDV_ is reduced in MI patients compared with healthy, age-matched controls. Secondly, MI patients with LVT demonstrated reduced wash-in of blood to the distal LV during late diastole, detected by the prominent drop of A-wave KE from the mid-ventricle to the apex. This parameter of LV blood flow disturbance was most strongly associated with the presence of LVT.

### Precursor to LV flow stasis

Blood stasis in the LV is the hallmark of LVT formation. In the detailed mapping of LV flow KE, we noted that the global LV KE parameters are significantly altered in health vs. MI patients. LV KEi_EDV_ averaged over the complete cardiac cycle, systolic KEi_EDV_ and peak E-wave KEi_EDV_ were all significantly reduced in patients with MI. Even though these global LV flow KE parameters were not significantly different in patients with/without thrombus, they are likely to be the key initial substrates of flow stasis post-MI.

### Reduced and delayed diastolic wash-in of the LV

During early and late diastole, blood flow into the LV cavity takes very little time due to intra-ventricular pressure gradients. In this study, the TD showed an increase from healthy controls to LVT− and further to LVT+ patients, demonstrating that patients with LVT have significantly delayed wash-in of the LV.

Additionally, the relative drop of distal A-wave KE was significantly higher in MI patients with LVT vs. patients without LVT. This finding suggests that there is a reduction in distal intra-ventricular pressure gradients due to a relative increase in distal (apical) pressures within the LV cavity in patients with LVT and that the late filling phase of diastole plays an important role in reduced wash-in of the LV.

The in-plane KE of blood flow was significantly higher in MI patients with LVT than those without LVT. Our data support the notion that an increase in the in-plane flow will reduce the proportion of through-plane flow in the LV cavity, and thus less blood will pass through the ventricle per-unit-time resulting in reduced global wash-in and wash-out of the LV. In addition to lower wash-in and wash-out, such non-physiological in-plane flow may exert strain on the LV wall, resulting in more dilatation and increase in endothelial dysfunction in the endocardium, similar to the vascular system.^16^ An increased in-plane rotational component of the intra-cavity LV flow may also increase the shear stress on the platelets and activate them, which would promote thrombosis.^17^

### Traditional risk factors

Akin to published studies in patients with LVT, our study also demonstrated the association of infarct location and depressed EF to LVT.^2^^,^^18^^,^^19^ However, this study failed to associate infarct size with presence of LVT (*P* = 0.82). This is possibly because we included patients with chronic infarction in both the MI groups. In chronic infarcts, the infarct size substantially decreases from the acute stage, which may lessen the overall impact of infarct size. In addition, even though there was a trend of apical regional wall motion score to be higher in LVT− patients, this study did not demonstrate any significant changes in LVT+ patients. This may be explained by the fact that in a previous study by Keren *et al.*,^20^ none of the inferior MI patients had thrombus, whereas this study recruited 12.5% inferior/posterior MI who had LVT. In this study, MI patients with diabetes were more likely to have LVT (*P* < 0.01). This finding is likely due to under representation of patients with diabetes in the LVT− MI cohort as observational studies have demonstrated prevalence of diabetes is around 24–36% in MI.^21^

### LVT characteristics and associated flow changes

LVT volume was the only parameter which had some association to flow characterisation (minimal KE, in-plane KE, and TD flow parameters). Mobility, produrance, and murality of the thrombus did not demonstrate any significant flow association. We speculate this may be because thrombus characteristics change rapidly after MI and probably depend on the timing of the imaging.

### Limitations

This study was a prospective cohort study, hence our results cannot be used to determine the prevalence of thrombus in MI. Additionally, we studied differences in flow patterns in the presence of LVT and not prior to its genesis, and a prospective evaluation of the parameters tested in this study is required. Arrhythmias can introduce errors in 4D flow analysis. To reduce these errors, we performed robust quality checks on all the data. Additionally, we used retrospectively gated acquisition sequence for 4D flow to reduce time blurring.^14^ The LV geometry was defined by LV cine stack which was done using breath-hold technique while the 4D flow was done using free breathing. Hence, although spatial miss-registration was corrected for, other issues still remain including difference in heart rate and physiological conditions. This may have impact on the time-varying flow characteristics which could not be corrected for. The temporal resolution of the 4D flow was 40 ms, which may affect the overall quality of TD assessment and make them less reliable.

## Conclusions

This study provides mechanistic insights into disturbed flow patterns in MI patients with and without thrombus. Patients with MI have reduced global LV KE and MI patients with LVT have evidence of reduced wash-in of the LV. Among all imaging biomarkers, the relative drop of distal intra-ventricular A-wave KE, which represents the distal late-diastolic wash-in of the LV, was most strongly associated with the presence of LVT. Future studies need to evaluate the prognostic significance of blood flow KE changes in the LV in patients with LVT.

## Supplementary Material

Supplementary Data
