# Corrigendum to: Left ventricular thrombus formation in myocardial infarction is associated with altered left ventricular blood flow energetics

**DOI:** 10.1093/ehjci/jey148

**Published:** 2018-10-01

[*Eur Heart J Cardiovasc Imaging* 2019;**20**:108–17]

There were three errors in the originally published version:

1. Affiliation 3 was incorrect in the original version and should have read ‘Radboudumc, Department of Cardiology, Geert Grooteplein Zuid 10, 6525 GA Nijmegen, The Netherlands.’

2. Under the heading ‘CMR examination’, the abbreviation ‘MR’ has been spelt out as ‘mitral regurgitation’ but should have read ‘magnetic resonance’.

3. Under the heading ‘Haemodynamic analysis’, the abbreviation ‘MR’ stands for ‘mitral regurgitation’.

