# Supplementary material for: Left ventricular thrombus formation in myocardial infarction is associated with altered left ventricular blood flow energetics
Source: Eur Heart J Cardiovasc Imaging. 2018 Aug 22;20(1):108–17. doi: 10.1093/ehjci/jey121 (PMC6302263; doi:10.1093/ehjci/jey121)
Supplement: Supplementary Data [file jey121_supplementary_data.docx]

**Supplementary Material**

**Contents**

Supplementary Methods

1. Detailed CMR protocol
2. 4D flow data reconstruction method
3. 4D flow error corrections and quality checks
4. Image Analysis
5. Intra-/inter-observer reliability checks
6. Statistical analysis
   1. Intra-/inter-observer reliability tests
   2. Power calculations

**Supplementary Results**

1. Intra-/inter-observer reliability checks

**Tables**

1. S Table 1. 4D flow echo-planar imaging (EPI) sequence details.
2. S Table 2. Detailed intra-/inter-observer tests
3. S Table 3. Inter-rater reliability of main KE parameter thresholds
4. S Table 4. Transmurality of apical scar in LVT- and LVT+ groups.
5. S. Table 5. Association of LV thrombus characteristics to LV flow KE parameters in 36 patients with LVT.

**Figures**

1. S Figure 1. *CMR* protocol.

**CMR protocol (S Figure 1)**

CMR was performed on dedicated cardiovascular 1.5 Tesla Philips Ingenia system equipped with a 28-channel coil and Philips dStream digital broadband MR architecture technology. LGE-imaging was only done in patients as per previous published methods [1].

The CMR protocol was as follows:

1. Survey images

2. The following cines were defined using survey: VLA, HLA, 3-chamber (LVOT-views), and the LV volume contiguous short axis stack. All cines were acquired with a balanced steady-state free precession (bSSFP), single-slice breath-hold sequence. Typical parameters for bSSFP cine were as follows: SENSE factor 2, flip angle 60°, echo time (TE) 1.5 milliseconds, repetition time (TR) 3 milliseconds, field of view 320-420 mm according to patient size, slice thickness 8 mm, and 30 phases per cardiac cycle.

3. Contrast injection (Magnevist: 0.2 mmol/kg) followed by early gadolinium enhancement (EGE) imaging (3 of 5 SAX slices, 2-chamber and 4-chamber). EGE sequence details are as follows: Inversion recovery spoiled gradient echo (GE) sequence with sense factor of 1.7, TE/TR of 2.8/5.7msec, flip angle 25° and a slice thickness of 8mm.

4. Look-Locker to determine the T1-inversion time

5. LGE imaging at 15-minutes from gadolinium-based contrast injection. LGE-imaging was done by phase sensitive inversion recovery (PSIR) spoiled gradient echo (GE) sequence. PSIR sequence details are as follows: SENSE factor 1.7, typical TE/TR of 3.0/6.1msec, flip angle of 25°, slice thickness of 10 mm and with Look-Locker scout determined TI.

6. For whole heart 4D flow, field of view (FOV) was planned in trans-axial plane making sure whole heart was in FOV. If necessary number of slices was increased. 4D flow was done using FFE pulse sequence (EPI based, 3D) with retrospective ECG-triggering (EPI sequence is detailed in S. Table 1).

**4D flow data reconstruction** **method**

4D flow encoding was performed by standard 4-point encoding. Online/offline 4D flow data quality assurance checks were done as per previous published literature[2].

**4D flow error corrections and quality checks**

The effects of concomitant gradient terms were compensated using Maxwell correction methods by the CMR scanner. Remaining background errors were corrected by the local phase correction (LPC) filter on the CMR scanner performed in two-dimensional way- slice by slice. The LPC is a magnitude-weighted spatial low pass filter; pixels that are expected to be part of the static background are used with a higher weight than noisy background pixels or pixels that are expected to contain flow to determine the local phase offset. LPC uses surrounding tissue to determine “static” areas[3,4].

All three-directional phase contrast data sets were investigated for phase aliasing artefacts. If present then phase unwrapping was performed as per previously published guidelines on phase-contrast methods[5]. Additionally, any spatial misalignment of 4D flow data to cine imaging was corrected before any flow analysis was performed. This was done by visualizing streamlines in 4-chamber view at peak systole and repositioning them over descending aorta. Similar checks were done during diastole in 4-chamber and 2-chamber views for peak mitral inflow streamlines.

**Image Analysis**

Left ventricular endocardial contours were derived for all temporal phases from the short-axis cine acquisition using semi-automated image segmentation using dedicated software and left ventricular volumes and EF were obtained using standard methods at the core lab. Apical regional wall motion (RWM) was scored as per previous published methods: 1=normal; 2=hypokinetic; 3=akinetic and 4=diskinetic[6].

Presence of LVT on EGE imaging, infarct location and its size on LGE imaging were performed according to published guidelines[7]. LVT was characterised as mural, mobile or protruding on cines. LVT was contoured manually on LGE images to compute its volume.

**Diastolic flow assessment**

Mitral valve (MV) stroke volume (SV) was calculated using validated techniques including retrospective valve tracking, with measurement planes positioned perpendicular to the inflow direction on 2- and 4-chamber cines[8]. Contour segmentation was performed manually. Peak E-wave and A-wave velocity averaged for full inflow area were recorded.

**Intra-/inter-observer reproducibility tests**

For inter-observer tests, PG and RVG contoured SAX LV cine volumetric stack in 20 random study subjects blinded to each other’s analysis. Automated KE parameters were again generated using the new endocardial contours. For intra-observer tests, PG re-analysed LV SAX cines after 3-months. Akin to inter-observer tests, automated KE parameters were generated using the new endocardial contours.

**Statistical Analysis**

**Intra-/inter-observer reproducibility tests**

Bland-Altman analysis was done to investigate bias (accuracy, %), upper and lower of agreements (%, precision). In addition, Spearman's rank correlation coefficient was investigated for all KE parameters. Weighted kappa analysis was used to investigate categorical thresholds for inter-rater agreement.

**Sample Size Calculations**

Sample size calculations were performed by Machin et al’s methods[9]. Since this was a first mechanistic study, limited know-how existed on which KE parameter would demonstrate significant differences within the defined study population. To inform the recruitment strategy, we used published KE values in heart failure patients[10]. Assuming a similar reduction in systolic KE in patients with LVT (mean difference of 2μJ/ml from healthy subjects) as in heart failure patients and assuming a standard variation of 2μJ/ml within the three groups, a sample size of at least 30 in each of the three groups (n=90) would give a power of 80% at an alpha of 0.05.

**Results**

**Intra-/inter-observer reliability checks**

Overall, global LV KE parameters demonstrated very low bias (intra: average 2%; inter: average 4%) and good precision (intra: -16% to 20%; inter: -21% to 13%) (S. Table 2). KE drop from base to mid-ventricle had modest bias but low precision. However, KE drop from mid-ventricle to apex demonstrated better bias (intra: average 1%; inter: average 2%) and precision (intra: -11% to 10%; inter: -12% to 15%). Assessment of rotation KE of the LV demonstrated minimal bias (intra: 1%; inter:0%) and reasonable precision (intra: -18% to 17%; inter: -19% to 19%).

Inter-rater reliability of main KE parameters thresholds were good (in-plane KE propagation >37%; weighted-kappa=1, A-wave KE drop >85% from mid to apex; weighted-kappa=0.63 and PT from base to mid >31msec; weighted-kappa=0.67) (S. Table 3).

**Reference:**

1. Garg P, Kidambi A, Foley JRJ, Musa T Al, Ripley DP, Swoboda PP, Erhayiem B, Dobson LE, McDiarmid AK, Greenwood JP, Plein S. Ventricular longitudinal function is associated with microvascular obstruction and intramyocardial haemorrhage. Open Hear. 2016 Jan 1;3(1):e000337.

2. Garg P, Westenberg JJM, van den Boogaard PJ, Swoboda PP, Aziz R, Foley JRJ, Fent GJ, Tyl FGJ, Coratella L, ElBaz MSM, van der Geest RJ, Higgins DM, Greenwood JP, Plein S. Comparison of fast acquisition strategies in whole-heart four-dimensional flow cardiac MR: Two-center, 1.5 Tesla, phantom and in vivo validation study. J Magn Reson Imaging. 2017 May 4;47(1):272–81.

3. Walker PG, Cranney GB, Scheidegger MB, Waseleski G, Pohost GM, Yoganathan AP. Semiautomated method for noise reduction and background phase error correction in MR phase velocity data. J Magn Reson Imaging. 3(3):521–30.

4. MacDonald ME, Forkert ND, Pike GB, Frayne R, Drangova M, Fenster A. Phase Error Correction in Time-Averaged 3D Phase Contrast Magnetic Resonance Imaging of the Cerebral Vasculature. Zhang H, editor. PLoS One. 2016 Feb 24;11(2):e0149930.

5. Lotz J, Meier C, Leppert A, Galanski M. Cardiovascular Flow Measurement with Phase-Contrast MR Imaging: Basic Facts and Implementation. RadioGraphics. 2002 May;22(3):651–71.

6. Keren A, Goldberg S, Gottlieb S, Klein J, Schuger C, Medina A, Tzivoni D, Stern S. Natural history of left ventricular thrombi: their appearance and resolution in the posthospitalization period of acute myocardial infarction. J Am Coll Cardiol. 1990 Mar 15;15(4):790–800.

7. Cerqueira MD, Weissman NJ, Dilsizian V, Jacobs AK, Kaul S, Laskey WK, Pennell DJ, Rumberger JA, Ryan T, Verani MS. Standardized myocardial segmentation and nomenclature for tomographic imaging of the heart. A statement for healthcare professionals from the Cardiac Imaging Committee of the Council on Clinical Cardiology of the American Heart Association. Circulation. 2002 Jan 29;105(4):539–42.

8. Roes SD, Hammer S, van der Geest RJ, Marsan NA, Bax JJ, Lamb HJ, Reiber JHC, de Roos A, Westenberg JJM. Flow assessment through four heart valves simultaneously using 3-dimensional 3-directional velocity-encoded magnetic resonance imaging with retrospective valve tracking in healthy volunteers and patients with valvular regurgitation. Invest Radiol. 2009 Oct;44(10):669–75.

9. Machin D, Campbell M, Fayers, P PA. Sample Size Tables for Clinical Studies. Second Ed Blackwell Sci IBSN. 1997;(IBSN 0-86542-870-0):18–20.

10. Kanski M, Arvidsson PM, Töger J, Borgquist R, Heiberg E, Carlsson M, Arheden H, Mosterd A, Hoes A, Stewart S, MacIntyre K, Hole D, Capewell S, McMurray J, Mcmurray J, Adamopoulos S, Anker S, Auricchio A, Böhm M, Dickstein K, Eriksson J, Bolger A, Ebbers T, Carlhäll C, Carlsson M, Töger J, Kanski M, Bloch K, Ståhlberg F, Heiberg E, Arvidsson P, Töger J, Heiberg E, Carlsson M, Arheden H, Eriksson J, Dyverfeldt P, Engvall J, Bolger A, Ebbers T, Carlhäll C, Töger J, Kanski M, Carlsson M, Kovács S, Söderlind G, Arheden H, Gharib M, Rambod E, Kheradvar A, Sahn D, Dabiri J, Pasipoularides A, Redfield M, Jacobsen S, Burnett J, Mahoney D, Bailey K, Rodeheffer R, Kanski M, Töger J, Steding-Ehrenborg K, Xanthis C, Bloch K, Heiberg E, Heiberg E, Sjögren J, Ugander M, Carlsson M, Engblom H, Arheden H, Carlsson M, Heiberg E, Töger J, Arheden H, Heiberg E, Engblom H, Engvall J, Hedström E, Ugander M, Arheden H, Rathi V, Doyle M, Yamrozik J, Williams R, Caruppannan K, Truman C, Fulford A, Hunt S, Abraham W, Chin M, Feldman A, Francis G, Ganiats T, Pedrizzetti G, Canna G, Alfieri O, Tonti G, Eriksson J, Carlhäll C, Dyverfeldt P, Engvall J, Bolger A, Ebbers T, Pasipoularides A, Shu M, Ashish S, Womack M, Glower D, Zajac J, Eriksson J, Dyverfeldt P, Bolger A, Ebbers T, Carlhäll C-J, Frisard M, Broussard A, Davies S, Roberts L, Rood J, Jonge L, Carlsson M, Andersson R, Bloch K, Steding-Ehrenborg K, Mosén H, Stahlberg F, Maceira A, Cosín-Sales J, Roughton M, Prasad S, Pennell D. Left ventricular fluid kinetic energy time curves in heart failure from cardiovascular magnetic resonance 4D flow data. J Cardiovasc Magn Reson. 2015 Dec 20;17(1):111.

**Tables**

**S Table 1.** 4D flow echo-planar imaging (EPI) sequence details

| Acceleration method | Parallel imaging; sense factor 2 in phase-encode AP direction  EPI factor of 5 |
| --- | --- |
| Flip-angle | 10 º |
| VENC (cm/s) | 150 |
| FOV (mm) | 350-400 |
| TE (ms) | 3.5 |
| TR (ms) | 10 |
| Partial k-space coverage in phase- encoding directions | 90% |
| Signal averages | 1 |
| ECG gating | Retrospective |
| Respiratory compensation | Free-breathing |
| Number of slices |  |
| Acquired temporal resolution (ms) | 4×TR = 40 |
| Reconstructed number of phases | 30 |
| In-plane spatial resolution (acquired) | 3×3×3mm (isotropic) |
| In-plane spatial resolution (reconstructed) | 2×2×3mm |

**S Table 2.** Intra and inter observer tests for reliability of kinetic energy parameters.

|  | Intra-observer tests | | | | | | Inter-observer tests | | | | | |
| --- | --- | --- | --- | --- | --- | --- | --- | --- | --- | --- | --- | --- |
|  | **Bias** | | **LL** | **UL** | **Correl.** | **P value** | | **Bias** | **LL** | **UL** | **Correl.** | **P value** |
|  | **Global LV Kinetic Energy** | | | | | | | | | | | |
| LV KEi_EDV_ | 1 | -8 | | 10 | 1.00 | 0.97 | | -3 | -15 | 10 | 0.99 | 0.90 |
| Minimal KEi_EDV_ | 5 | -23 | | 32 | 0.96 | 0.86 | | -6 | -33 | 21 | 0.96 | 0.79 |
| Systolic KEi_EDV_ | -2 | -24 | | 20 | 0.98 | 0.97 | | -1 | -21 | 18 | 0.98 | 0.92 |
| Diastolic KEi_EDV_ | 3 | -15 | | 21 | 0.99 | 0.94 | | -4 | -20 | 12 | 0.99 | 0.91 |
| Peak E-wave KEi_EDV_ | 5 | -11 | | 20 | 0.99 | 0.84 | | -4 | -18 | 9 | 0.99 | 0.85 |
| Peak A-wave KEi_EDV_ | 3 | -12 | | 19 | 0.99 | 0.88 | | -5 | -21 | 11 | 0.99 | 0.79 |
|  | **Relative KE drop from base to apex** | | | | | | | | | | | |
| E-wave (B→M) | -1 | -109 | | 107 | 0.91 | 0.84 | | 17 | -56 | 90 | 0.83 | 0.85 |
| A-wave (B→M) | -9 | -112 | | 94 | 0.67 | 0.68 | | 10 | -90 | 110 | 0.28 | 0.40 |
| E-wave (M→A) | 0 | -6 | | 7 | 0.86 | 0.89 | | 1 | -8 | 9 | 0.77 | 0.70 |
| A-wave (M→A) | -1 | -16 | | 13 | 0.91 | 0.83 | | 2 | -16 | 21 | 0.84 | 0.65 |
|  | **In-plane KE proportion (%)** | | | | | | | | | | | |
| LV KE | -1 | -14 | | 12 | 0.99 | 0.97 | | 1 | -15 | 16 | 0.99 | 0.97 |

Bias, LL (lower limit of agreement) and UL (upper limit of agreement) expressed in percentage.

**S Table 3.** Inter-rater reliability of main KE parameter thresholds.

| KE parameter | Criteria | Inter-rater agreement | Weighted Kappa |
| --- | --- | --- | --- |
| In-plane KE Proportion | >37% | 100% | 1 |
| A-wave KE drop (M→A) | >85% | 85% | 0.63 |
| TD (base to mid) | >31msec | 85% | 0.67 |

**S. Table 4.** Transmurality of apical scar in LVT- and LVT+ groups.

| AHA Segment | Transmurality (%) of scar in LVT- group | | Transmurality (%) of scar in LVT+ group | | *P-value |
| --- | --- | --- | --- | --- | --- |
|  | **Median** | **IQR** | **Median** | **IQR** |  |
| 13th | 43 | 53 | 36 | 30 | 0.55 |
| 14th | 31 | 63.3 | 49 | 41 | 0.23 |
| 15th | 31 | 34 | 28 | 26 | 0.85 |
| 16th | 18 | 32 | 22 | 42 | 0.55 |
| *Mann-Whitney Test | | | | | |

**S. Table 5.** Association of LV thrombus characteristics to LV flow KE parameters in 36 patients with LVT.

| CMR parameters | Mobile | Mural | Protruding | Volume |
| --- | --- | --- | --- | --- |
| N (%) / mean ± standard deviation | 6 (17%) | 26 (72%) | 10 (27%) | 4.9±10* ml/m2 |
| LV KEi_EDV_ | 0.136 | -0.048 | 0.048 | -0.141 |
| Minimal KEi_EDV_ | 0.23 | 0 | 0 | 0.355 (0.03) |
| Systolic KEi_EDV_ | 0.265 | -0.101 | 0.101 | -0.082 |
| Diastolic KEi_EDV_ | -0.029 | 0.042 | -0.042 | -0.074 |
| Peak E-wave KEi_EDV_ | -0.036 | -0.006 | 0.006 | -0.233 |
| Peak A-wave KEi_EDV_ | 0.287 | -0.233 | 0.233 | -0.256 |
| Rotational in-plane KE (%) | 0.022 | -0.018 | 0.018 | 0.355 (0.03) |
| E-wave (B→M) | -0.115 | 0.173 | -0.173 | 0.302 |
| A-wave (B→M) | 0.05 | -0.113 | 0.113 | -0.115 |
| E-wave (M→A) | -0.036 | 0.066 | -0.066 | -0.094 |
| A-wave (M→A) | 0.022 | -0.042 | 0.042 | -0.031 |
| TD (B→M) | -0.057 | 0.051 | -0.051 | 0.334 (0.04) |

All values are ***Spearman rank correlation coefficient***. Only significant P-values expressed in brackets. *indexed volume

**Figures**

**
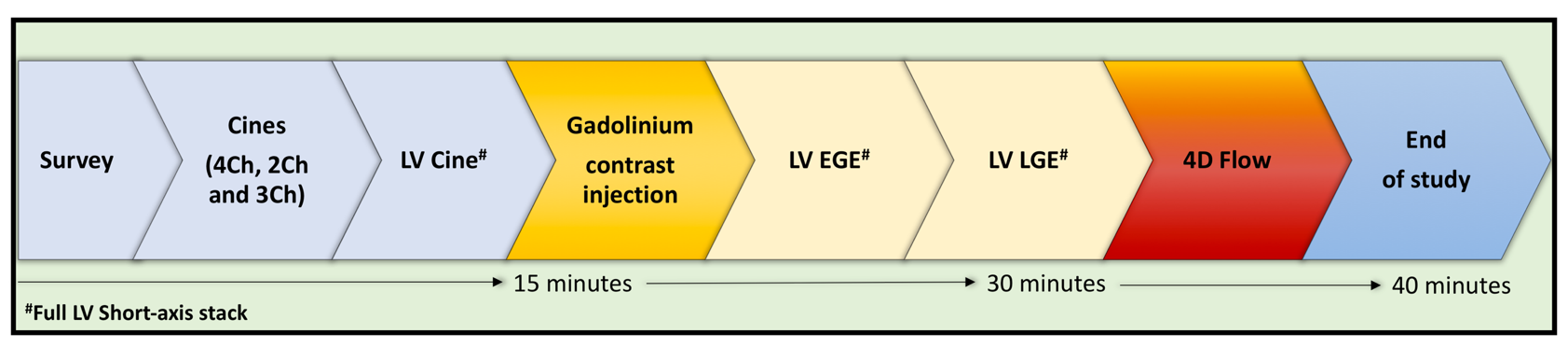
S. Figure 1.** CMR protocol. Total scanning time was 40-45minutes.
